# Prospective study on chronic kidney disease and the prevalence of pruritus among internal medicine patients

**DOI:** 10.6026/973206300212905

**Published:** 2025-08-31

**Authors:** Sachin Puthusseriyil Shaji, Yash Sharan, Yashita Dharni, Mona Mushtaque, Anjaly Mecheril Chandran, Shameela Farha, Rahil Abdusamad Nainam Valappil

**Affiliations:** 1 Department of Emergency Medicine, General Hospital, Muvattupuzha, Kerala, India; 2 Department of Anesthesia and Critical Care, Lok Nayak Hospital, New Delhi, India; 3 Department of General Medicine, Adesh Institute of Medical Sciences and Research (AIMSR), Bathinda, Punjab, Chandigarh, India; 4 Department of Gastroenterology, Malankara Orthodox Syrian Church Medical Mission Hospital, Kolenchery, Kerala, India; 5 Department of Emergency Medicine, Calicut Hospital & Nursing home, Calicut, Kerala, India; 6 Department of Critical care, Iqraa International Hospital, Calicut, Kerala, India

**Keywords:** Chronic kidney disease, pruritus, uremic itch, quality of life, prospective study

## Abstract

The prevalence and severity of pruritus over six months is assessed among 124 internal medicine patients diagnosed with varying stages of chronic kidney disease (CKD). Pruritus was observed in over half the cohort, with increasing frequency in advanced CKD stages. Severity was correlated with elevated serum urea and phosphorus levels. Sleep disturbance and reduced quality of life were common among affected individuals. Thus, we show the clinical relevance of pruritus in CKD management.

## Background:

Chronic kidney disease (CKD) is a progressive condition affecting millions worldwide, often accompanied by a range of distressing complications that reduce quality of life [1]. Chronic kidney disease-associated pruritus (CKD-aP) is a common problem in patients with kidney disease, especially in those who are receiving dialysis. Among these, pruritus commonly referred to as uremic itch, is a frequently overlooked yet highly prevalent symptom, particularly in patients with moderate to advanced renal dysfunction [2, 3]. Pruritus in CKD is thought to result from complex interactions involving uremic toxin accumulation, systemic inflammation, secondary hyperparathyroidism and dysregulation of opioid receptors [4]. Despite being non-life-threatening, pruritus significantly affects patient comfort, sleep quality, psychological well-being and overall health-related quality of life [5]. In clinical practice, it remains underdiagnosed and inadequately managed, especially in early CKD stages or in non-dialysis patients under general internal medicine care [6]. Most studies to date have focused on dialysis populations, leaving a gap in knowledge regarding pruritus prevalence and characteristics across all CKD stages [7]. Therefore, it is of interest to evaluate the prevalence, severity and clinical correlates of pruritus among internal medicine patients with CKD, thereby guiding more proactive screening and management approaches.

## Materials and Methods:

This prospective observational study was conducted over a period of six months in the internal medicine department of a tertiary care hospital. A total of 124 adult patients (aged 18 years and above) diagnosed with chronic kidney disease (CKD) stages 2 to 5, based on estimated glomerular filtration rate (eGFR) as per KDIGO guidelines, were enrolled consecutively. Patients on dialysis or with dermatological conditions unrelated to CKD (e.g., eczema, psoriasis, scabies) were excluded. Detailed clinical evaluations were performed at baseline, including medical history, current medications and laboratory parameters such as serum creatinine, urea, calcium, phosphorus, parathyroid hormone (PTH) and hemoglobin levels. The presence of pruritus was assessed using a standardized questionnaire and severity was graded using the 10-point Visual Analog Scale (VAS). Patients reporting a VAS score ≥4 were considered symptomatic. Additional information was collected on sleep quality, skin changes and interference with daily activities. All participants were followed monthly and any new onset or worsening of symptoms was documented. Statistical analysis involved chi-square tests for categorical variables and t-tests for continuous variables. Logistic regression was used to identify independent predictors of pruritus. Ethical approval was obtained from the institutional review board and all participants provided informed consent prior to enrollment.

## Results:

Pruritus was found to be highly prevalent among patients with chronic kidney disease, particularly in those with advanced stages (4 and 5). Approximately 58% of the study population reported pruritus, with higher severity scores associated with increased serum urea, phosphorus levels and reduced eGFR. Table 1 (see PDF) shows that the study population was well distributed across CKD stages, with a slight predominance of males and older adults. Table 2 (see PDF) shows most patients belonged to CKD stages 3 to 5, allowing analysis of pruritus across varying renal function levels. Table 3 (see PDF) shows over half of the participants reported pruritus, most commonly in CKD stage 4 and 5 patients. Table 4 (see PDF) shows Moderate to severe pruritus was predominantly found in advanced CKD stages. Table 5 (see PDF) shows Patients with pruritus showed significantly higher mean urea and phosphorus levels. Table 6 (see PDF) shows higher pruritus prevalence was associated with significantly reduced eGFR. Table 7 (see PDF) shows sleep disturbance was commonly reported among those with moderate to severe pruritus. Table 8 (see PDF) shows dry skin was the most frequently associated dermatological finding among pruritic patients. Table 9 (see PDF) shows antihistamines were the most commonly used therapy, though with limited effectiveness. Table 10 (see PDF) shows multiple biochemical parameters were independent predictors of pruritus in CKD.

## Discussion:

This prospective study demonstrates a substantial prevalence of pruritus in internal medicine patients with chronic kidney disease (CKD), with nearly 58% of the cohort affected. The burden of pruritus was closely associated with worsening renal function, as evident by significantly lower estimated glomerular filtration rate (eGFR) and higher serum urea and phosphorus levels in affected individuals [8]. These findings are consistent with the proposed pathophysiological mechanisms of uremic pruritus, including accumulation of metabolic toxins, secondary hyperparathyroidism and systemic inflammation [9]. The high frequency in CKD stages 4 and 5 aligns with existing evidence, but the presence of pruritus even in earlier stages (stage 3) highlights the importance of early symptom recognition and management [10]. Pruritus in this study significantly impacted patients' sleep and quality of life, particularly in the physical and psychological domains, emphasizing its clinical relevance beyond discomfort [11]. The lack of consistent and effective treatment was notable; commonly used therapies such as antihistamines and emollients provided only partial relief, underscoring the need for more targeted pharmacological and non-pharmacological interventions, including neuromodulators like gabapentin or non-opioid receptor antagonists [12]. While diabetes and age were not independent predictors in this cohort, logistic regression confirmed that high serum urea, phosphorus and lower eGFR independently predicted the presence of pruritus. These markers could serve as practical clinical indicators to stratify pruritus risk and guide early intervention [13]. Given the underreporting of symptoms by patients and under recognition by clinicians, routine screening for pruritus in CKD patients should be incorporated into internal medicine and nephrology practice [14]. Overall, this study highlights that pruritus is not just a benign symptom but a significant contributor to disease burden and reduced well-being in CKD patients [15]. Early diagnosis, comprehensive symptom assessment and individualized treatment protocols are critical to improving patient outcomes [16]. Further research is needed to develop more effective therapies and to explore the molecular mechanisms behind CKD-associated pruritus in non-dialysis populations.

## Conclusion:

Pruritus is a common and clinically significant complication in chronic kidney disease, affecting over half of internal medicine patients, especially in advanced stages. Its presence correlates with biochemical derangements like elevated urea and phosphorus, reduced eGFR and substantially impairs sleep and quality of life. Early recognition and targeted intervention strategies are essential for improving symptom control and patient well-being in this under-recognized aspect of CKD care.
